# Spatio-temporal analysis of host preferences and feeding patterns of malaria vectors in the sylvo-pastoral area of Senegal: impact of landscape classes

**DOI:** 10.1186/1756-3305-6-332

**Published:** 2013-11-19

**Authors:** El Hadji Malick Ngom, Jacques-André Ndione, Yamar Ba, Lassana Konaté, Ousmane Faye, Mawlouth Diallo, Ibrahima Dia

**Affiliations:** 1 Unité d’Entomologie Médicale, Institut Pasteur de Dakar, 36 Avenue Pasteur, Dakar, BP 220, Sénégal; 2 Laboratoire d’Ecologie Vectorielle et Parasitaire, Université Cheikh Anta Diop de Dakar, Dakar, Senegal; 3 Centre de Suivi Ecologique, Dakar, Senegal

**Keywords:** *An. gambiae* s.l, Trophic preferences, Anthropophilic rates, Landscape classes, Blood meals, Pastoral area

## Abstract

**Background:**

The study of vector feeding behaviour is an important step in the understanding of the epidemiology of vector borne diseases. The main objective of this work was to study the spatio-temporal host preferences and blood-feeding patterns of malaria vectors in a pastoral area of Senegal where cattle breeding is the main human activity.

**Methods:**

Malaria vectors were collected indoors by pyrethrum spray catch in 16 villages belonging to 4 different landscape classes (wooded savanna, shrubby savanna, bare soils and steppe). Blood meals sources were determined using a direct enzyme-linked immunosorbent assay (ELISA).

**Results:**

The blood meal origins of 1886 freshly fed *An. gambiae* s.l. were determined. Among these blood meals, most were taken on a single host: 40.1% on human and 37.1% on animal. The range in proportions of blood meals taken from human were 25–62.4% in wooded savanna villages, 23.5–61.9% in shrubby savanna villages, 31.3–70% in bare soils villages and 57.7–68.7 in steppe villages. Blood meals taken from bovines were very heterogeneous with two clusters localized in the Northeast and Southwest axis of the study area that corresponds to the distribution of the main water ponds. Patent mixed blood meals taken from human and non-human were significantly higher than those taken from two animals, the highest proportions being observed in September (shrubby savanna, bare soils and steppe villages) or October (wooded savanna villages).

**Conclusions:**

These observations suggest that in this pastoral area, differences in feeding patterns of malaria vectors are merely linked to the specific localization of villages and are not influenced by landscape class distribution. In addition, the temporal variations in the anthropophilic rates are influenced by the presence of standing water in the study area.

## Background

For many vector-borne diseases, the vector biting behaviour is an important factor in the epidemiology of the disease they transmit. Knowledge on the blood-feeding habits is important for implementation of effective vector control strategies [1]. In the case of malaria, the frequencies of *Anopheles* bites on humans affect the likelihood that a mosquito in contact with a carrier of gametocytes becomes infected and able to transmit human *Plasmodium*. This probability is more important for vectors that exclusively bite only humans and reflects the degree of human-vector contact, which can be used in the implementation and evaluation of the impact of control measures [2].

Even though malaria is a non-transmissible pathogen between humans and animals, domestic animals can have a significant role in its epidemiology through the attraction or repulsion they may have on the vectors. Thus, in the prevention of transmission, domestic animals living in close proximity to humans can attract anopheline vectors and thereby reduce the transmission of *Plasmodium*[3]. The interaction between humans and alternative hosts can be more pronounced in areas with a high concentration of livestock, particularly in pastoral areas. The presence of livestock in close proximity to human habitations may produce different trophic profiles including human or animal only or mixed blood meals from both [4]. This is the case in the main pastoral area of Senegal, where entomological studies showed heterogeneous trophic preferences of malaria vectors [5]. In this area livestock breeding is essentially traditional with a seasonal dynamic under the control of nomadic pastoralism largely linked to water and pasture availability. In fact, during the dry season, the lack of pasture and water lead to the concentration of cattle near the water boreholes and incite some herdsmen to leave the area. During the rainy season, they move back, thus increasing (in addition to the transhumant farmers from others regions of Senegal) substantially the population size of the livestock. This situation associated with the climatic conditions favourable for local vectors increase the risk of the emergence of vector-borne diseases such as Rift Valley and West Nile fevers that are endemic in this area [6], but might also affect malaria transmission.

The use of domestic animals living together with human populations to divert the blood-seeking vectors from human hosts has been suggested as an effective strategy for malaria control [7]. Studies conducted in Kenya have shown that the proportion of malaria vectors with human blood meals was significantly lower in areas with higher concentrations of domestic animals [8,9] while Seyoum *et al*. [10] showed that the presence of cattle in homesteads tends to increase the man biting rates of malaria vectors. Therefore, the mosquitoes blood-feeding behaviour may be largely influenced by environmental factors in addition to their innate features [11]. Taking into account the spatial distribution of villages and hamlets as well as landscape classes, we analyzed the spatio-temporal host preferences and blood-feeding patterns of malaria vectors in the pastoral area of Ferlo, where cattle breeding is the main human activity.

## Methods

### Presentation of the study area

**Figure 1:**
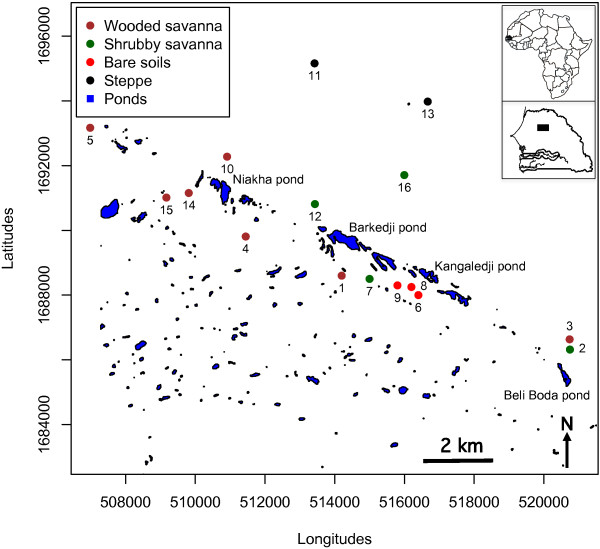
Localisation of the study villages.

This study was conducted in 16 villages within the Sahelian region in the Sylvo-pastoral area of Senegal (Figure 1) from July to November 2009. The climate is typically Sahelian with a summer monsoon (the rainy season) that lasts from July to mid-October. Overall, the mean annual rainfall is mainly provided by squall lines, and ranges from 300 mm to 500 mm. During the rainy season, a large quantity of small and temporary ponds is thus formed leading to an environment favouring mosquito breeding. These ponds are widely distributed, some isolated, and others organized in clusters of all sizes (Figure 1). The main known ponds are presented in Figure 1 (Niakha, Barkedji, Kangaledji and Beli Boda).

The population of this area is estimated at 14,200 inhabitants comprising of Fulani (85%), Wolof (12%), Moors and Serer (3%). They grow mainly millet during the rainy season but cattle breeding is one of the main economic activities. Livestock including cattle, sheep, goats, poultry and equines (donkeys and horses) are the main domestic animals. Malaria transmission is seasonal in the study area and is transmitted predominantly by *An. arabiensis* ([5,12], Dia *et al*., unpublished data).

During the dry season, farmers and their herds are concentrated near the water boreholes that remain the only available water points during that period whereas during the rainy season, the tranhumance from all breeding regions of Senegal bring a large number of farmers and their cattle into this region in search of pasture and water surfaces for livestock needs.

Within the study area, four different landscape classes were defined using remote sensing and geospatial analyses from a SPOT 5 satellite image based on the description of the vegetation classes according to the combination of the FAO [13] and CSA [14] systems. All 16 sampling sites were geo-referenced with a hand-held GPS receiver and each of them was classified to the corresponding landscape class (Figure 1, Table 1).

**Table 1:** Main characteristics of the study sites

**N° sites**	**Villages**	**Latitude N**	**Longitude W**	**Land cover type**
1	Barkedji	15°17′	14°53′	Wooded savanna
2	Keur Alpha Goudal	15°14′	14°47′	Shrubby savanna
3	Keur Racine Sow	15°15′	14°47′	Wooded savanna
4	Dague Nabe	15°17′	14°52′	Wooded savanna
5	Diabal	15°18′	14°56′	Wooded savanna
6	Keur Dadal Sow	15°16′	14°51′	Bare soil
7	Keur Adama Sow	15°16′	14°51′	Shrubby savanna
8	Keur Aliou Diallo	15°15′	14°50′	Bare soil
9	Keur Gallo Sow	15°16′	14°51′	Bare soil
10	Keur Adama	15°17′	14°53′	Wooded savanna
11	Keur Bandji	15°19′	14°52′	Steppe
12	Keur Demba	15°17′	14°52′	Shrubby savanna
13	Keur Diallo	15°19′	14°50′	Steppe
14	Niakha	15°17′	15°54′	Wooded savanna
15	Niakha Ndiaybe	15°15′	14°50′	Wooded savanna
16	Wouro Samba Kibel	15°17′	14°51′	Shrubby savanna

### Mosquito sampling and processing

Adults resting mosquitoes were collected using the Pyrethrum Spray Catch method in selected bedrooms from July to November 2009. Upon collection, anopheline vectors were sorted, counted and morphologically identified following the key of Gillies & de Meillon [15]. Blood meals from blood-fed mosquitoes were collected onto filter paper for subsequent determination of the host source. The origin of blood meals was identified using an enzyme-linked immunosorbent assay (ELISA) according to Beier *et al*. [16] using peroxidase-conjugated antibodies (Kirkegaard and Perry, Gaithersburg, MD). The choice of antibodies was made by taking into account the prevalent domestic animals present in the study villages. Thus, five different antibodies were used: anti-human, anti-bovine, anti-ovine, anti-chicken and anti-equine. All blood meals sources were determined simultaneously using the five antibodies.

### Data analysis

Data were entered into Microsoft Excel and analysed using the R Gui software (v.3.0.2). The proportions of blood meals taken on each of the five vertebrate hosts tested were estimated by the percentage of the number of blood meals taken on each host over the total number of blood meals identified.

The anthropophilic rates were calculated as the proportion of the mosquitoes that fed on humans out of the total blood meals determined. For each study site, the proportions of patent mixed blood meals were calculated as the proportion of mosquitoes fed at least on two different vertebrate hosts over the total blood meals identified. The chi-squared test was used to compare the proportions of blood meals whereas analysis of variance (ANOVA) was used to compare the mean proportions of blood meals between landscape classes.

To study the influence of landscape classes on trophic preferences, the proportions of blood meals between sites belonging to the same landcape class were compared and pooled to assess their association with the trophic profile of the vectors.

## Results

A total of 1886 blood meals from *An. gambiae* s.l. resting females were tested by ELISA (Table 2). Most of these blood meals were taken on a single host (human or animal). The percentage of non-reacting blood meals was estimated to be 8.4% whereas 14.4% of blood meals were taken on two different vertebrate hosts. All the blood meals sampled from the bare soil landscape were identified. The proportions of non-identified blood meals were statistically comparable between the three other landscape classes (*χ*^2^ = 4.8, df = 2, p = 0.09).

**Table 2:** **Number and percentage of *An. gambiae *** **resting females fed on each of the 5 vertebrate hosts tested in the study villages**

**Landscape classes/villages**			**Single blood meals**					**Mixed blood meals**		
		**Number tested**	**Human**	**Bovine**	**Ovine**	**Chicken**	**Equine**	**Animal/Animal**	**Human/Animal**	**Undetermined**
Wooded savanna										
	Barkedji	675	237 (35.1)	90 (13.3)	57 (8.4)	6 (0.9)	139 (20.6)	0 (0)	77 (11.4)	69 (10.2)
	Keur Racine Sow	80	28 (35)	7 (8.8)	9 (11.3)	0 (0)	11 (13.8)	0 (0)	3 (3.8)	22 (27.5)
	Dague Nabe	134	79 (59)	4 (3)	9 (6.7)	0 (0)	10 (7.5)	0 (0)	20 (14.9)	12 (9.0)
	Diabal	141	88 (62.4)	3 (2.1)	6 (4.3)	0 (0)	24 (17)	0 (0)	13 (9.2)	7 (5.0)
	Keur Adama	26	8 (30.8)	2 (7.7)	8 (30.8)	0 (0)	3 (11.5)	0 (0)	3 (11.5)	2 (7.7)
	Niakha	120	43 (35.8)	33 (27.5)	8 (6.7)	8 (6.7)	0 (0)	0 (0)	7 (5.8)	21 (17.5)
	Niakha Ndiaybe	308	77 (25)	104 (33.8)	9 (2.9)	5 (1.6)	29 (9.4)	1 (0.3)	78 (25.3)	5 (1.6)
Shrubby savanna										
	Keur Alpha Goudal	136	32 (23.5)	44 (32.4)	9 (6.6)	0 (0)	8 (5.9)	0 (0)	30 (22.1)	13 (9.6)
	Keur Adama Sow	21	13 (61.9)	3 (14.3)	0 (0)	0 (0)	4 (19)	0 (0)	1 (4.8)	0 (0)
	Keur Demba	25	12 (48)	0 (0)	2 (8)	1 (4)	0 (0)	0 (0)	9 (36)	1 (4)
	Wouro Samba Kibel	42	26 (61.9)	0 (0)	5 (11.9)	0 (0)	4 (9.5)	0 (0)	7 (16.7)	0 (0)
Bare soil										
	Keur Dadal Sow	17	11 (64.7)	2 (11.8)	1 (5.9)	0 (0)	1 (5.9)	0 (0)	2 (11.8)	0 (0)
	Keur Aliou Diallo	16	5 (31.3)	5 (31.3)	0 (0)	0 (0)	3 (18.8)	0 (0)	3 (18.8)	0 (0)
	Keur Gallo Sow	20	14 (70)	3 (15)	0 (0)	0 (0)	1 (5)	0 (0)	2 (10)	0 (0)
Steppe										
	Keur Bandji	26	15 (57.7)	0 (0)	0 (0)	0 (0)	6 (23.1)	0 (0)	3 (11.5)	2 (7.7)
	Keur Diallo	99	68 (68.7)	4 (4)	1 (1)	0 (0)	8 (8.1)	0 (0)	14 (14.1)	4 (4)
Total		1886	756 (40.1)	304 (16.1)	124 (6.6)	20 (1.1)	251 (13.3)	1 (0.1)	272 (14.4)	158 (8.4)

(): Percentage (%).

### Trophic preferences

Within the study area, the majority of blood meals were of human (40.1%) origin followed by bovine (16.1%). The remaining blood meals were from equines (13.3%), ovine (6.6%) and chicken (1.1%).

**Figure 2:**
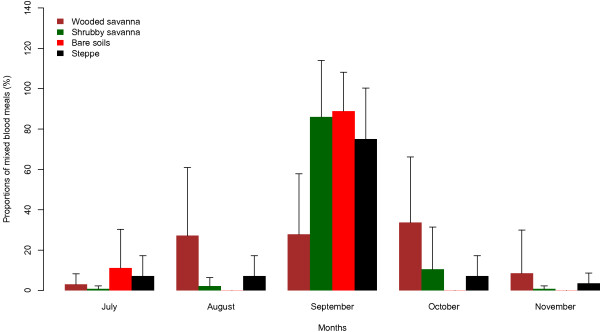
Temporal variations of mixed blood meals (Human/Animal) type within each of the four landscape classes identified in the study area.

Only one patent mixed blood meal was taken on two different non-human hosts in a village of wooded savanna (Niakha), while mixed blood meals taken from human and non-human host were more frequent and were observed in all villages (Table 2). The highest proportions of these mixed blood meals were observed in September (shrubby savanna, bare soil and steppe villages) or October in wooded savanna villages (Figure 2). No significant difference was observed between the number of patent mixed blood meals between the four landscape classes for each month (F_3,12_ = 0.71, p = 0.6 for July, F_3,12_ = 2.2, p = 0.1 for August, F_3,12_ = 3.4, p = 0.8 for September, F_3,12_ = 0.9, p = 0.5 for October, F_3,12_ = 0.7, p = 0.6 for November).

**Figure 3:**
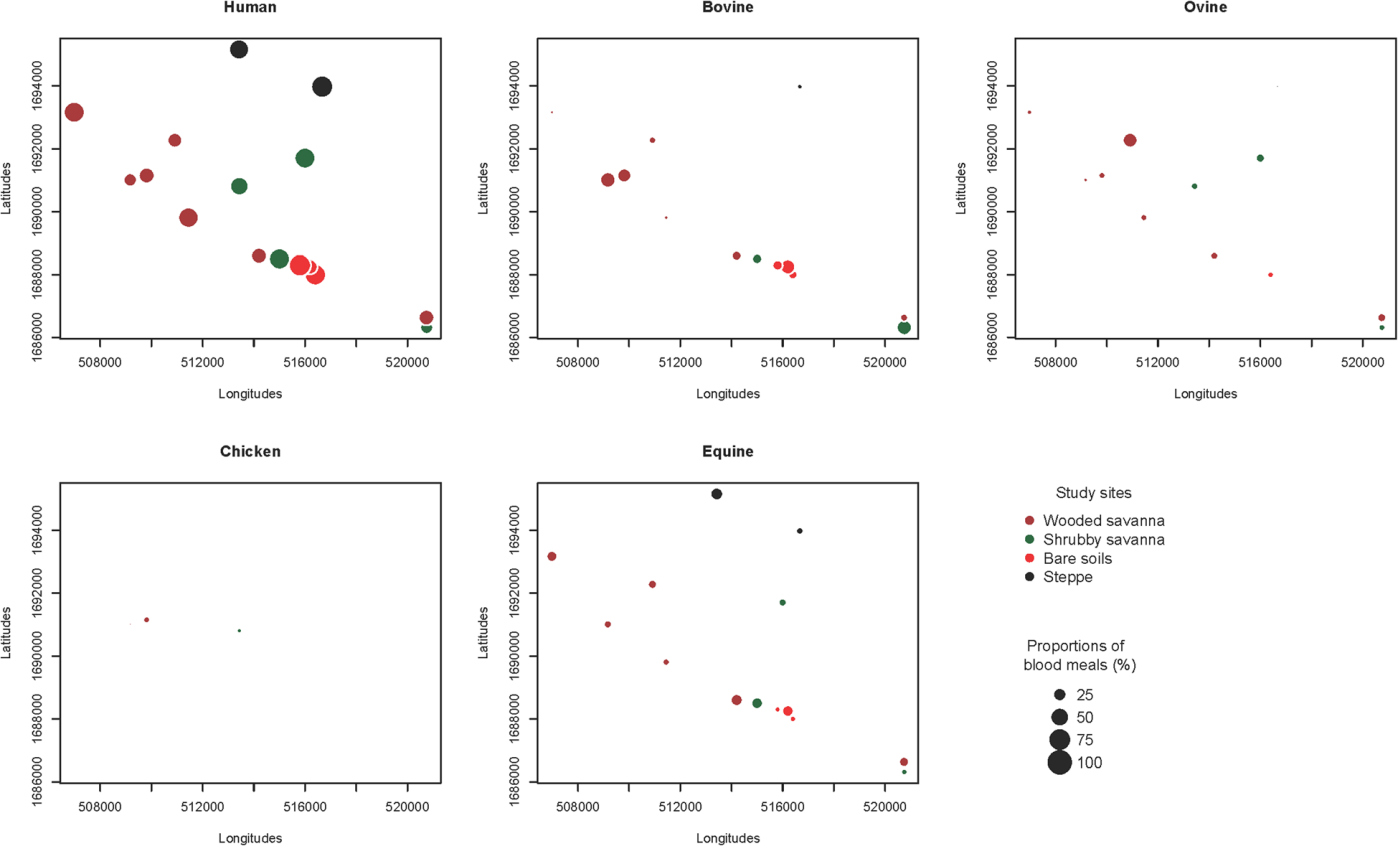
Spatial variations of the proportions of blood meals taken from each of the five vertebrate hosts in the study villages.

Spatially, in almost all villages, blood meals taken from humans were predominantly observed. Blood meals taken from bovine were very heterogeneous and seem to constitute two clusters localized respectively around Kangaledji and Niakha ponds (Figure 3). The proportions of blood meals taken from ovine were relatively low (between 1 and 31%) and were observed in 12 out of the 16 villages (Figure 3). Blood meals from chicken were observed only in two wooded savanna villages (Barkedji and Niakha) whereas blood meals from equines were observed in all villages (except in two villages from wooded savanna and 1 village from shrubby savanna).

### Anthropophilic rates

The range of the proportions of blood meals taken from humans were 25–62.4%, 23.5–61.9%, 31.3–70% and 57.7–68.7% respectively in wooded savanna, shrubby savanna, bare soils and steppe villages. These variations were statistically different between wooded savanna villages (*χ*^2^ = 86.4, df = 6, p < 0.0001) and shrubby savanna villages (*χ*^2^ = 28.6, df = 3, p < 0.0001) but neither between bare soils villages (*χ*^2^ = 6.1, df = 3, p = 0.11) nor between steppe villages (*χ*^2^ = 0.7, df = 1, p = 0.41).

**Figure 4:**
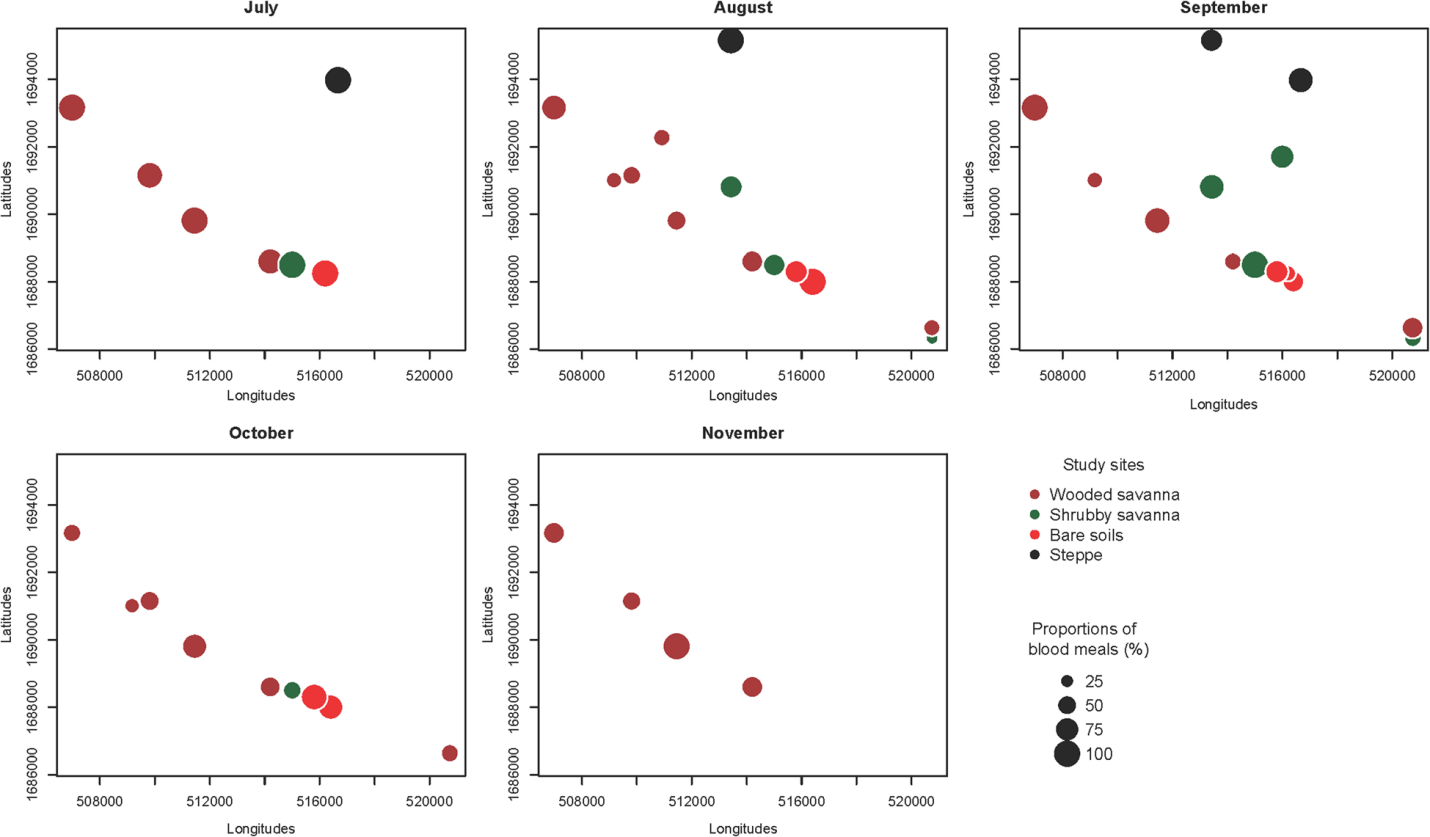
Temporal variations of the anthropophilic rates in each of the the study villages.

The anthropophilic rates were heterogeneous in July at the beginning of the rainy season (Figure 4). The highest proportions (range 90–100%) were observed only in 7 villages (4 in wooded savanna, 2 in shrubby savanna, 1 both in steppe and bare soils). It was mainly during the rainy season (August and September) that the blood meals taken from human were widespread and homogenously distributed in the study area. The anthropophilic rates were higher in September than in August (range 33.3–83.3; 34.1–97.8% respectively in August and September in wooded savanna villages, 20–66.7% in August and 37.3–100% in September in Shrubby savanna villages). For villages from bare soil and steppe, the differences were less marked but the blood meals taken from humans were more uniformly distributed in September (Figure 4).

At the end of the rainy season, the anthropophilic rates were low in shrubby savanna villages and bare soil whereas no blood meal from humans was observed in steppe villages in November. In wooded savanna villages, the anthropophilic rates increased in October (between 30 and 79.3%) and November (between 46.6 and 100%).

## Discussion

The evaluation of mosquito vectors host preferences is difficult under natural conditions and depends on the sampling techniques used [17]. Besides mosquito species with a highly specific feeding behaviour, there are other vectors whose feeding habits depend largely on the presence and accessibility of alternative hosts [18]. Thus, to avoid and minimize the potential bias due to the sampling method used, and to be able to compare the feeding habits of mosquitoes between villages and landscape classes, blood fed females were collected in human houses and far away from storehouses, pens and other livestock shelters.

Our results showed that single blood meals on human or animal were predominantly observed in comparison to patent mixed blood meals. In each of the four landscape classes, blood meals taken from humans were more frequently observed. The proportions of these blood meals could be underestimated as the ELISA method used is unable to detect multiple cryptic blood meals on humans whereas the inclusion of the frequencies of these blood meals may contribute to a better estimation of the entomological inoculation rate [19,20]. Inversely, the existence of single blood meals on animals could be a barrier to malaria transmission [21]. For this reason many human communities have intentionally used cattle near or inside human habitations in order to divert mosquitoes from humans to cattle [22].

On the other hand, almost all patent mixed blood meals were taken from humans and animals with observed proportions up to 36% in a shrubby savanna village. This may contribute to a reduction in transmission through a loss of *Plasmodium*. Indeed, Muriu *et al*. [3] suggested that during mixed feeding in non-human hosts, a loss of certain number of sporozoites is expected and could be of importance in malaria control. Such phenomenon would be more marked in wooded savanna villages and to a lesser extent in shrubby savanna villages where the highest proportions of mixed blood meals were observed. This situation may indicate that mixed herds are rare in the study area and/or human populations live in close proximity with livestock. Overall, the highest proportions of these mixed blood meals were observed in September in shrubby savanna, bare soil and steppe villages or in October in wooded savanna villages. This period corresponds to the period of highest mosquitoes densities in the area ([5,23], Dia *et al*. Unpublished data). This observation could be the result of hosts defence reactions against high vector abundance as suggested by Boreham & Garrett-Jones [24]. Therefore, several feedings are necessary in the same gonotrophic cycle to achieve complete repletion.

Depending on the villages, most of the single blood meals were taken from humans, equines and to a lesser extent from ovines. Previous studies on trophic preferences in the Barkedji village have shown the predominance of human and bovine blood meals [5]. Although less widely distributed, the proportions of blood meals from bovine were relatively important and associated with bare soil villages (south-western part of the study area) and two wooded savanna villages (Niakha and Niakha Ndiaybe situated in the north-western part of the study area). Indeed, the bare soil landscape in this zone, is where the livestock is preferentially parked including big ruminants (around Kangaledji pond). The two villages from wooded savanna (Niakha and Niakha Ndiaybe) are settled near one of the biggest water ponds in this area (Niakha pond), that constitutes the main source of water for humans as well as livestock. Therefore it is expected that mosquitoes that took their blood meals from these animals, rest inside human houses in these two villages.

The more widely distributed blood meals from humans and ovines can be explained by the availability of these hosts whatever the village or landscape classes considered. In fact, in the majority of houses in wooded savanna villages, small ruminants including sheep and goats are kept inside human habitations. The low proportions of blood meals taken from chicken are the result of a rarity of this host and/or a reduced attraction of the vectors as already shown in the area [5] or elsewhere in Senegal [25].

Our data exhibit widely dispersed blood meals taken from humans during August and September in the study area. During this period water and pasture are available for livestock. Thus, most of the cattle herds are left far away from human habitations, reducing thereby the contacts with human hosts and the possibility of blood meals on livestock. In October and November, the distribution of human blood meals is restricted to the villages situated along the north-western and south-eastern axis that correspond to the distribution chain of the main residual water ponds in the area in this period. Our observations suggest, therefore, that the observed differences are merely due to the specific localization of villages and are not influenced by the types of landscape classes. In addition, the presence of wide grazing surfaces around villages of steppe, bare soil and shrubby savanna keep animals distant to the human environment, while in wooded savanna villages, livestock usually live in close proximity with humans in the domestic and peridomestic environment.

## Conclusions

This study has demonstrated that in this pastoral area, malaria vectors show heterogeneous trophic preferences linked to the specific localization and seem not to be influenced by the distribution of landcape classes. It has further revealed that the temporal variations of malaria vector anthropophilic rates are influenced by the presence of standing water in the study area. Hence, any strategy for controlling malaria vectors in this area should take into account this heterogeneity for better control measure choices and implementation.

## Competing interests

The authors declare that they have no competing interests.

## Authors’ contributions

ID, LK, OF and MD designed and supervised the study. ID, EMN, JAN and YB performed field and laboratory activities. ID, EMN, MD analyzed the data, drafted and revised the manuscript. All authors revised and approved the final version of the manuscript.
